# An oncolytic HAdV-5 with reduced surface charge combines diminished toxicity and improved tumor targeting

**DOI:** 10.1016/j.omton.2024.200909

**Published:** 2024-11-23

**Authors:** Frederik Wienen, Robin Nilson, Ellen Allmendinger, Sarah Peters, Thomas F.E. Barth, Stefan Kochanek, Lea Krutzke

**Affiliations:** 1 Department of Gene Therapy, Ulm University, 89081 Ulm, Germany; 2 Department of Clinical Chemistry, Ulm University Medical Center, 89081 Ulm, Germany; 3 Institute of Pathology, Ulm University Medical Center, 89081 Ulm, Germany

**Keywords:** MT: Regular Issue, oncolytic virus, adenovirus, surface charge, hepatotoxicity, tumor targeting, systemic application, Hexon, intravenous, adenoviral vector

## Abstract

Human adenovirus type 5 (HAdV-5)-based oncolytic viruses hold significant promise for anti-cancer therapy. However, poor tumor-targeting and off-target organ transduction after systemic administration limit their therapeutic efficacy. In addition, the strong liver tropism of HAdV-5-based vectors poses the risk of hepatotoxicity. By genetic modification of the major capsid protein hexon we generated a HAdV-5-based oncolytic vector (HAdV-5-HexPos3) with reduced negative surface charge. Coxsackie and adenovirus receptor (CAR) binding-ablated (ΔCAR) HAdV-5-HexPos3_ΔCAR exhibited superior and CAR-independent transduction of various cancer cell lines *in vitro*, further enhanced in the presence of HAdV-5 naive murine plasma. Upon intravenous administration into tumor-bearing immunodeficient NSG mice, replication-deficient HAdV-5-HexPos3_ΔCAR vector particles showed significantly reduced off-target organ tropism in all tissues analyzed, including the liver. Moreover, we detected a significantly increased intratumoral vector load for HAdV-5-HexPos3_ΔCAR, leading to a 29-fold elevated tumor-to-liver ratio compared with a control vector with unmodified hexon. Intravenous injection of a conditionally replicating hexon-unmodified control vector induced severe hepatotoxicity in tumor-bearing NSG mice, while a conditionally replicating HAdV-5-HexPos3_ΔCAR vector was well tolerated and resulted in intratumoral vector presence for up to 56 days. HAdV-5-HexPos3_ΔCAR represents a promising vector platform for the generation of HAdV-5-based oncolytic viruses with reduced systemic toxicity and improved therapeutic efficacy.

## Graphical abstract

**Figure:**
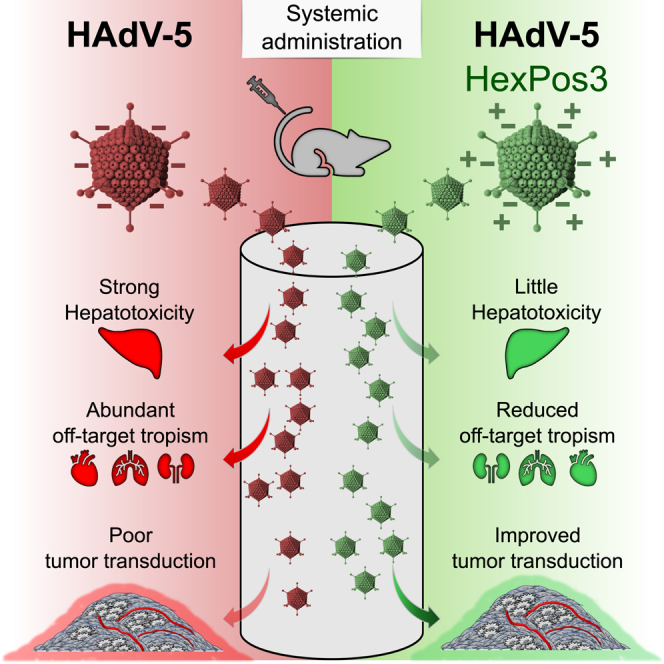

## Teaser

Kochanek and colleagues evaluate a genetically capsid-modified oncolytic adenovirus type 5 with reduced negative surface charge. The virus exhibited significantly reduced hepatotoxicity in tumor-bearing mice while tumor targeting was improved and intratumoral virus replication was confirmed. This virus represents a promising virus platform for future development of therapeutic oncolytic adenoviruses.

## Introduction

Oncolytic viruses are aimed to specifically infect and replicate in cancer cells, intended to induce cell lysis and evoke a tumor-directed activation of the immune system. Oncolytic adenoviral vectors (oAVs) based on human adenovirus type 5 (HAdV-5) are promising candidates; however, to date their clinical efficacy is still limited.^1^^,^^2^ Preferably, oAVs are administered intravenously (i.v.) to achieve a uniform intratumoral (i.t.) particle distribution and enable targeting of inaccessible tumors and metastases. To improve the therapeutic efficacy of HAdV-5-based oAVs, tumor transduction efficiency after i.v. administration needs to be enhanced and vector-induced toxicity due to off-target cell infection has to be reduced as much as possible.^3^

The inherent liver tropism of HAdV-5-based vectors represents a major obstacle with a majority of i.v. administered vector particles being rapidly sequestered by liver residential Kupffer cells (KCs), while remaining particles efficiently transduce hepatocytes.^4^^,^^5^^,^^6^^,^^7^^,^^8^ Therefore, high doses of i.v. injected HAdV-5 vectors may result in severe hepatotoxicity.^5^^,^^9^^,^^10^^,^^11^ Thus, reduction of the HAdV-5 liver tropism represents a key step to not only reduce vector toxicity but also to improved tumor targeting.

*In vitro*, binding of HAdV-5 to its primary receptor, the coxsackie and adenovirus receptor (CAR), represents the critical step for host cell infection.^12^*In vivo*, however, the HAdV-5 particle tropism is largely CAR independent.^13^^,^^14^ In contrast, hexon, the most abundant protein of the adenoviral capsid, is a major determinant for particle fate and distribution *in vivo*.^15^ Per viral particle, 720 hexon monomers assemble into 240 trimers, constituting the icosahedral capsid with its 12 vertices.^16^ Within hexon, three distinct loops (L1, L2, L4) can be identified, which together display a total of seven discrete hypervariable regions (HVR1-7),^17^ all of which are surface exposed.^18^ These HVRs are involved in various interactions of the particles with cellular and non-cellular host blood components.^19^ The HVRs significantly contribute to the net negative surface charge of the adenoviral particle,^20^ which may result in an electrostatic repulsion of the viral particle from the host cell surface and reduce vector infectivity. Moreover, the HVRs represent primary recognition sites for scavenger receptors (SRs) expressed on KCs.^6^^,^^21^ Blood coagulation factor X (FX) binds to HVR5 and HVR7^22^ and bridges the vector particle to heparan sulfate proteoglycans expressed on hepatocytes mediating cell transduction.^7^ Due to their surface exposure, HVRs further provide epitopes for antibodies and thus are involved in sequestration and neutralization of particles by the innate and adaptive immune system.^23^^,^^24^ Especially HVR1, the largest HVR within hexon, is predominantly composed of negatively charged amino acid residues. HVR1 is not only the preferred recognition site for neutralizing natural IgM antibodies but also mediates binding to SRs and thus significantly contributes to particle sequestration.^21^^,^^25^^,^^26^^,^^27^

Previously, we reported the generation of a genetically modified capsid-mutant HAdV-5 vector, in which a stretch of 13 predominantly negatively charged amino acids within HVR1 was replaced by 4 consecutive lysine residues.^28^ We showed that the resultant HAdV-5-HexPos3_ΔCAR vector exhibited a significantly reduced net negative surface charge and showed remarkably enhanced and CAR-independent transduction efficiency in human multipotent mesenchymal stromal cells (MSCs) and various cancer cell lines *in vitro*.^28^ Based on these findings, we considered HAdV-5-HexPos3 a promising candidate as an oncolytic vector.

In this work, we thoroughly characterized HAdV-5-HexPos3_ΔCAR regarding its oncolytic potential, safety, and anti-tumor efficacy *in vitro* and *in vivo*. HAdV-5-HexPos3 vector particles showed reduced off-target organ tropism accompanied by significantly reduced toxicity but improved tumor infection and i.t. replication upon i.v. administration in human xenograft tumor-bearing immunocompromised NOD SCID gamma (NSG) mice.

## Results

### HexPos3 capsid mutation significantly enhances transduction efficiencies of HAdV-5-based vectors *in vitro* but not after i.t. vector injection

**Figure 1:**
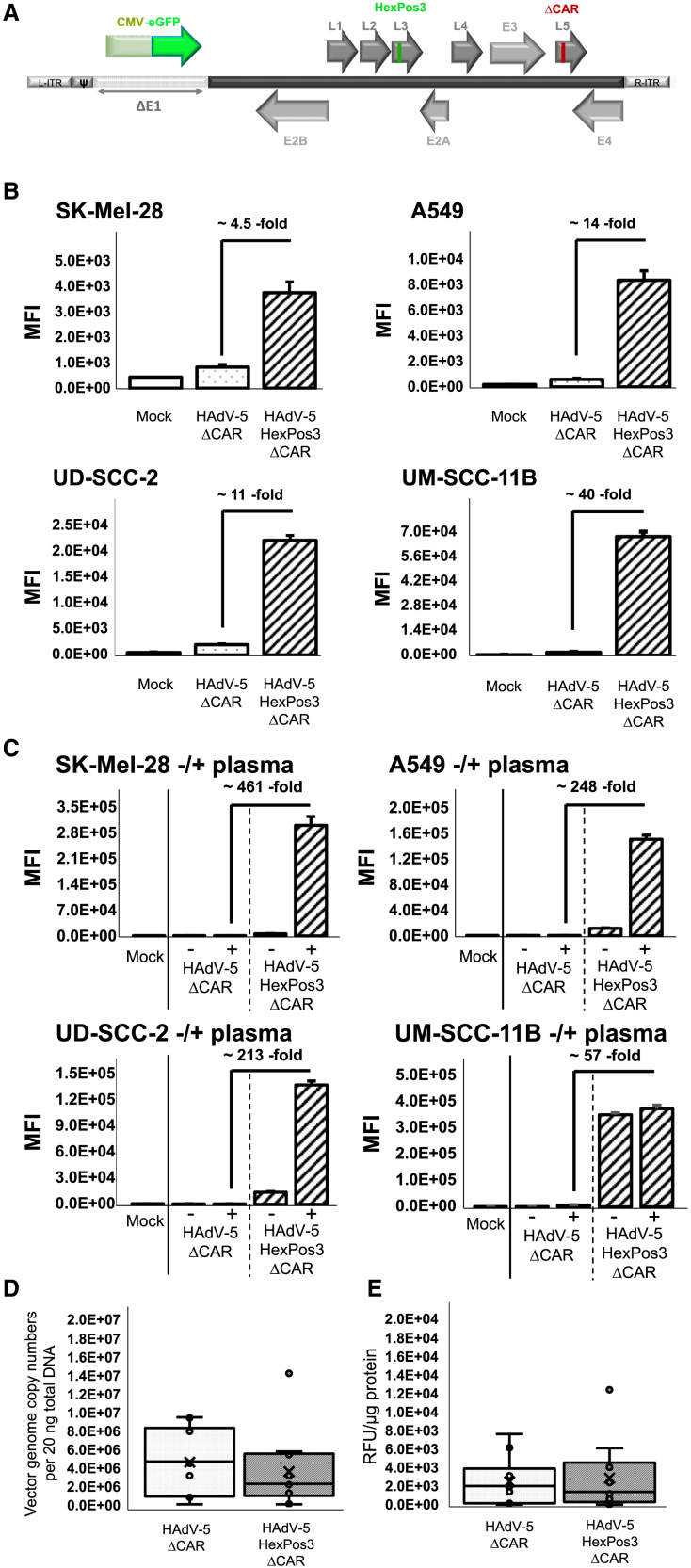
HAdV-5-HexPos3_ΔCAR exhibits improved transduction efficiencies *in vitro* but not after intratumoral vector injection *in vivo* (A) Schematic illustration about the genome arrangement of HAdV-5_ΔCAR and HAdV-5-HexPos3_ΔCAR. (B) SK-Mel-28, A549, UD-SCC-2, and UM-SCC-11B cells were transduced with the indicated vectors with pMOI of 1,000 and transduction efficiencies were evaluated 24 hpt by quantification of mean fluorescence intensities (MFI) using flow cytometric analysis. *n* = 3. (C) Indicated vectors were incubated in either PBS (−) or HAdV-5 naive NSG plasma (+) at room temperature for 10 min prior to transduction of SK-Mel-28, A549, UD-SCC-2, and UM-SCC-11B cells with pMOI of 1,000. Transduction efficiencies were evaluated 24 hpt by quantification of MFI using flow cytometric analysis. *n* = 3. (D and E) UD-SCC-2 tumor-bearing NSG mice were intratumorally injected with 1 × 10^10^ vector particles of either HAdV-5_ΔCAR or HAdV-5-HexPos3_ΔCAR after 28 days of tumor growth. *n* = 10. Three days after vector injection, mice were euthanized, and tumor transduction efficiencies were evaluated by (D) quantification of intratumoral vector genome copy numbers and eGFP expression levels using qPCR and (E) fluorometric analysis of tumor tissue homogenates. RFU, relative fluorescence units.

Using replication-deficient and CAR binding-ablated (ΔCAR) vector particles (for a schematic illustration of the applied genetic modifications see Figure 1A), we first performed *in vitro* transduction assays in SK-Mel-28, A549, UD-SCC-2, and UM-SCC-11B cells to confirm the enhanced transduction efficiencies of HAdV-5-HexPos3_ΔCAR as reported previously.^28^ Compared with the hexon-unmodified control vector HAdV-5_ΔCAR, HAdV-5-HexPos3_ΔCAR exhibited 4- to 40-fold enhanced transgene expression levels in all cancer cell lines tested (Figure 1B). As a next step, and with respect to the ensuing *in vivo* experiments, we analyzed whether murine blood plasma components altered the transduction efficiency of HAdV-5-HexPos3_ΔCAR. To this end, vector particles were pre-incubated in either PBS (−) or HAdV-5-naive NSG plasma (+) prior to cell transduction (Figure 1C). While transduction efficiencies of HAdV-5_ΔCAR remained rather unaffected by NSG plasma, transduction efficiencies of HAdV-5-HexPos3_ΔCAR were strongly increased in the presence of murine plasma (>450-, >200-, >200-, and >50-fold for SK-Mel-28, A549, UD-SCC-2, and UM-SCC-11B cells, respectively, compared with HAdV-5_ΔCAR) (Figure 1C).

Next, we investigated the oncolytic potential of HAdV-5-HexPos3_ΔCAR *in vivo*. We established subcutaneous UD-SCC-2 tumors in immune-deficient NSG mice, a human xenograft tumor model previously generated and thoroughly characterized in our laboratory.^29^^,^^30^ After 28 days of tumor growth we i.t. injected 1 × 10^10^ vector particles of either HAdV-5_ΔCAR or HAdV-5-HexPos3_ΔCAR and harvested tumors 3 days after vector injection. Tumor transduction efficiencies were evaluated by quantification of i.t. vector genome copy numbers (Figure 1D) and eGFP expression levels in tumor homogenates (Figure 1E). Surprisingly, and in contrast to previous *in vitro* data, both fluorometric and quantitative polymerase chain reaction (qPCR) analysis indicated similar, although not improved tumor transduction efficiencies of HAdV-5-HexPos3_ΔCAR compared with HAdV-5_ΔCAR (Figures 1D and 1E).

### HAdV-5-HexPos3_ΔCAR exhibits reduced off-target tropism and improved tumor targeting after single i.v. vector injection

To assess biodistribution patterns and tumor targeting of charge-modified HAdV-5-HexPos3_ΔCAR after i.v. vector administration, we injected 2 × 10^10^ particles of replication-defective HAdV-5_ΔCAR or HAdV-5-HexPos3_ΔCAR via the tail vein in UD-SCC-2 tumor-bearing NSG mice after 14 days of tumor growth. Three days later, vector loads in spleen, lung, kidney, heart, liver, and tumors were determined by qPCR analysis of vector genome copy numbers. Biodistribution data of HAdV-5_ΔCAR control vectors were obtained in parallel using in all regards the exact same experimental parameters. Although noteworthy, the dataset was used as a reference for another research project in our group and the respective data have already been published before.^29^ However, in the spirit of the 3R principle, control vector injections were not performed twice but the data used for both projects.

**Figure 2:**
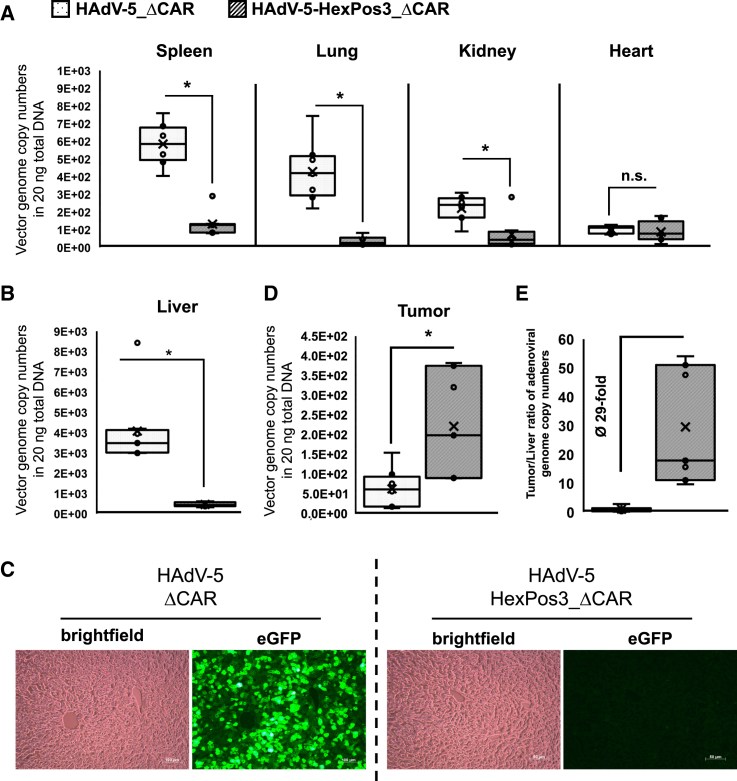
Significantly reduced off-target organ tropism and improved tumor targeting by HAdV-5-HexPos3_ΔCAR after intravenous injection UD-SCC-2 tumor-bearing NSG mice were intravenously injected with 2 × 10^10^ vector particles of replication-defective HAdV-5_ΔCAR or HAdV-5-HexPos3_ΔCAR after 14 days of tumor growth. Three days after vector injection, organs and tumors were harvested and vector genome copy numbers were assessed by qPCR. *n* = 7–8. ∗*p* < 0.05; n.s., not significant. (A) Spleen (∗*p* ≤ 0.00016; Wilcoxon test), lung (∗*p* ≤ 0.00023; Welch’s test), kidney (∗*p* ≤ 0.011; Wilcoxon test), and heart (n.s. *p* ≥ 0.48; Student’s t test). (B) Liver (∗*p* ≤ 0.00094; Wilcoxon test). (D) Tumor (∗*p* ≤ 0.021; Welch’s test). (E) Tumor/liver ratios. (C) Hepatocyte transduction by HAdV-5_ΔCAR and HAdV-5-HexPos3_ΔCAR was additionally evaluated with eGFP expression by fluorescence microscopy of corresponding liver sections.

Compared with HAdV-5_ΔCAR, we detected significantly reduced vector amounts of HAdV-5-HexPos3_ΔCAR in all organs analyzed except for the heart, in which comparable amounts of both vectors were found (Figure 2A). Most importantly, HAdV-5-HexPos3_ΔCAR was taken up significantly less by the liver (Figure 2B). We further confirmed these data by fluorescence microscopy of corresponding liver sections, which showed abundant eGFP-positive cells in liver sections of mice, injected with HAdV-5_ΔCAR, while almost no eGFP-positive cells were observed in liver sections of mice injected with HAdV-5-HexPos3_ΔCAR (Figure 2C). In contrast, we detected elevated i.t. vector amounts in mice injected with HAdV-5-HexPos3_ΔCAR compared with mice injected with HAdV-5_ΔCAR (Figure 2D). Given that and due to its almost completely ablated liver tropism, HAdV-5-HexPos3_ΔCAR showed a ∼29-fold improved tumor-to-liver ratio compared with HAdV-5_ΔCAR (Figure 2E).

### Generation and characterization of conditionally replicating adenoviral vectors

**Figure 3:**
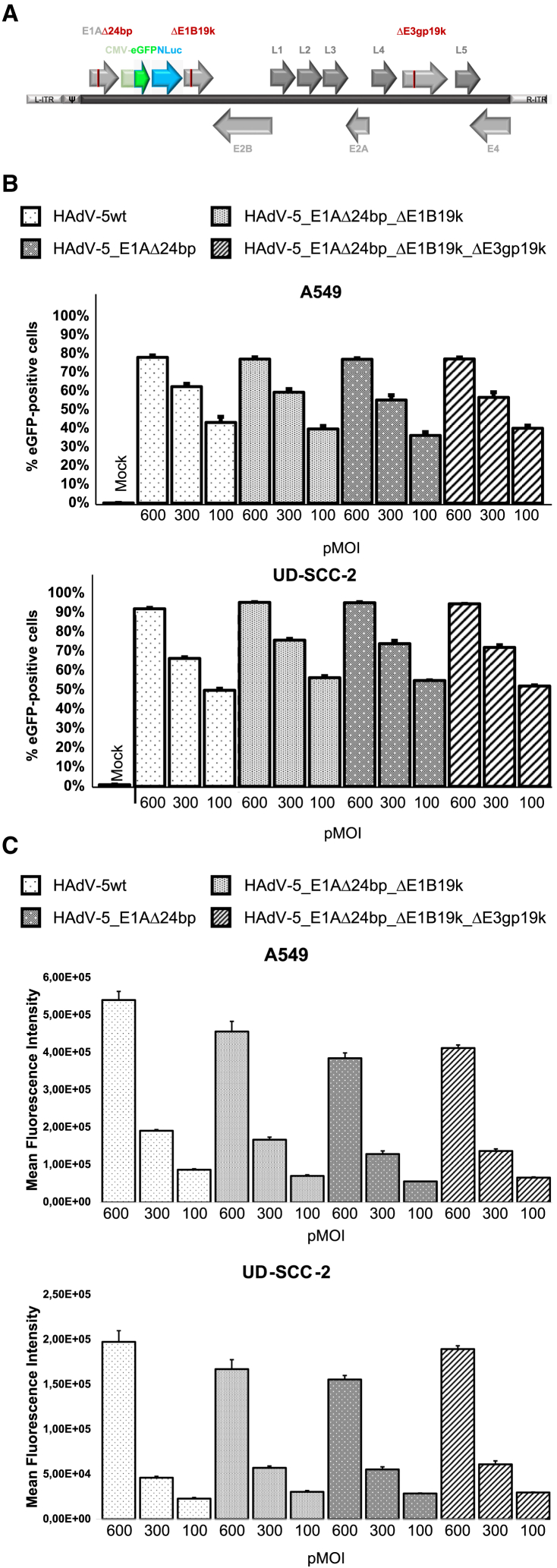
Generation of conditionally replicating adenoviral vectors Based on HAdV-5wt, three conditionally replicating adenoviral vectors (CRAds) were generated HAdV-5_E1AΔ24bp, HAdV-5_E1AΔ24bp_ΔE1B19k, and HAdV-5_E1AΔ24bp_ΔE1B19k_ΔE3gp19k. HAdV-5wt and all CRAds were equipped with the CMV promotor-driven eGFP and NanoLuciferase fusion expression cassette introduced between *E1A* and *E1B* open reading frames. (A) Schematic illustration of the applied mutations and their approximate localization within the linear double-stranded adenoviral DNA genome. L-/R-ITR, left/right inverted terminal repeats; ψ, packaging signal. (B and C) A549 and UD-SCC-2 cells were infected with HAdV-5wt or respective CRAds with the indicated pMOI. (B) eGFP-positive cells and (C) mean fluorescence intensities were quantified by flow cytometry 24 hpi. *n* = 3. pMOI, particle multiplicity of infection.

Tumor lysis by oAVs necessarily requires vector replication. In terms of safety, vector replication must be tightly restricted to malignant tumor cells while omitting healthy cells. Thus, we generated and compared three conditionally replication-competent adenoviral vectors (CRAds) based on the HAdV-5 wild-type (HAdV-5wt) virus. To this end, we consecutively introduced defined mutations into adenoviral regulatory genes known to be essential for virus replication in healthy cells; however, negligible in tumor cells (Figure 3A). Viruses additionally carried an eGFP-NanoLuciferase fusion reporter cassette that was introduced in a non-coding region of the adenoviral genome.

As a first mutation, we deleted a defined 24 bp stretch within the conserved region 1 (CR1) of *E1A* (HAdV-5_E1AΔ24bp) (Figure 3A), which results in a non-functional E1A protein that is unable to bind pRb, and thus fails to induce cell-cycle progression in healthy cells.^31^^,^^32^ As a second mutation, we deleted a defined 147 bp stretch within the *E1B19k* gene (HAdV-5_E1AΔ24bp_ΔE1B19k)^33^ (Figure 3A), which counteracts cellular apoptotic pathways by preventing oligomerization of the pro-apoptotic proteins BAK and BAX at the outer mitochondrial membrane.^34^^,^^35^^,^^36^^,^^37^ As a third mutation, we deleted parts of the *E3gp19k* gene (HAdV-5_E1AΔ24bp_ΔE1B19k_ΔE3gp19k) (Figure 3A), without affecting other *E3*-encoded viral proteins. During virus replication, E3gp19k binds and retains MHC class I proteins within the endoplasmic reticulum and thus prevents viral antigen presentation to cytotoxic T cells at the cell surface.^38^^,^^39^^,^^40^

HAdV-5wt and all CRAds were produced in A549 cells to normal titers. Transduction assays in A549 and UD-SCC-2 cells using different particle multiplicities of infection (pMOIs) for cell infection revealed similar eGFP expression levels, which indicated similar infectious titers and that applied genetic modifications did not interfere with vector infectivity (Figures 3B and 3C).

### Deletion of *E1B19k* improves the oncolytic potential of HAdV-5-based CRAds and restricts vector replication to cancer cells

**Figure 4:**
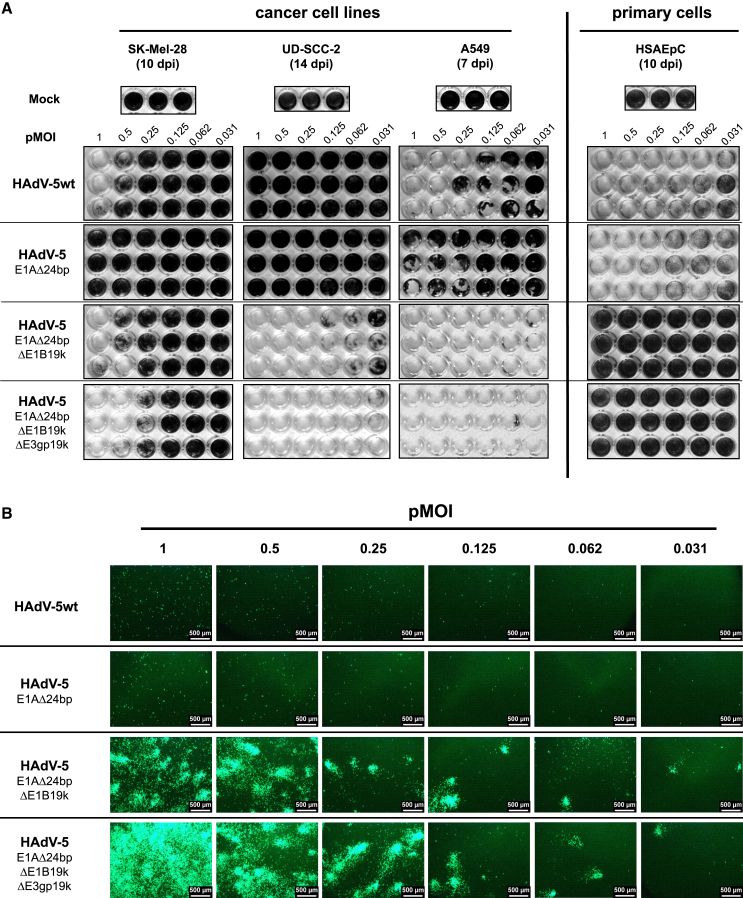
Deletion of E1B19k and E3gp19k restricts virus replication to cancer cells and improves the oncolytic potential of HAdV-5-based CRAds *in vitro* SK-Mel-28, UD-SCC-2, A549, and primary HSAEpC cells were infected with either HAdV-5wt, HAdV-5_E1AΔ24bp, HAdV-5_E1AΔ24bp_ΔE1B19k, or HAdV-5_E1AΔ24bp_ΔE1B19k_ΔE3gp19k with the indicated pMOI. (A) After the indicated days post infection (dpi), virus-induced cytotoxicity was analyzed by staining of remaining cells by crystal violet. (B) UD-SCC-2 cells were infected with HAdV-5wt, HAdV-5_E1AΔ24bp, HAdV-5_E1AΔ24bp_ΔE1B19k, or HAdV-5_E1AΔ24bp_ΔE1B19k_ΔE3gp19k at the indicated pMOI. After 10 days, eGFP-positive cells were visualized by fluorescence microscopy. *n* = 3–4. Scale bars, 500 μm.

To evaluate the oncolytic potential and cancer cell-restricted replication of CRAds, we performed *in vitro* cytotoxicity assays using SK-Mel-28, UD-SCC-2, and A549 cancer cell lines as well as primary HSAEpCs (human small airway epithelial cells) (Figure 4A). Cells were infected with either HAdV-5wt or the respective CRAds with low pMOI ranging from 1 to 0.031 in a serial 1:2 dilution. After the indicated time, vector spread and cell lysis was visualized by crystal violet staining of remaining cells.

HAdV-5_E1AΔ24bp lysed all tested cancer cell lines with lower efficiency than HAdV-5wt, indicating that the E1AΔ24bp mutation reduced the oncolytic potential of HAdV-5-based vectors (Figure 4A). Moreover, primary HSAEpCs were efficiently lysed by HAdV-5_E1AΔ24bp (Figure 4A), indicating that the E1AΔ24bp mutation alone did not restrict vector replication to cancer cells. In contrast, E1B19k-deleted vectors HAdV-5_E1AΔ24bp_ΔE1B19k showed significantly improved cell lysis compared with HAdV-5wt in all cancer cell lines tested (Figure 4A). Interestingly, the E3gp19k mutation further increased the effect, hence HAdV-5_E1AΔ24bp_ΔE1B19k_ΔE3gp19k vectors showed the most pronounced cytotoxic effect (Figure 4A). Most importantly, neither HAdV-5_E1AΔ24bp_ΔE1B19k nor HAdV-5_E1AΔ24bp_ΔE1B19k_ΔE3gp19k vectors replicated in primary HSAEpCs (Figure 4A), defining them as conditionally replicating vectors.

To visualize the vector spread, we evaluated UD-SCC-2 cells infected with different pMOIs of HAdV-5wt or respective CRAds by fluorescence microscopy (Figure 4B). Ten days after cell infection, only few eGFP-positive cells were observed in cells infected with HAdV-5wt or HAdV-5_E1AΔ24bp, indicative for absent or low-level vector replication. In contrast, HAdV-5_E1AΔ24bp_ΔE1B19k and HAdV-5_E1AΔ24bp_ΔE1B19k_ΔE3gp19k efficiently replicated and spread through UD-SCC-2 cells as indicated by comet-like shaped eGFP-positive cell clusters. Fitting to the results obtained by crystal violet staining, effects were even more pronounced for HAdV-5_E1AΔ24bp_ΔE1B19k_ΔE3gp19k vectors compared with HAdV-5_E1AΔ24bp_ΔE1B19k.

### The HexPos3 capsid mutation significantly reduces vector toxicities after single i.v. vector injection

Based on these findings, HAdV-5_E1AΔ24bp_ΔE1B19k_ΔE3gp19k, further referred to as human conditionally replicating adenoviral vector-5 (HCRAd-5), was equipped with the HexPos3 and the ΔCAR capsid modifications, resulting in HCRAd-5-HexPos3_ΔCAR. In addition, a firefly luciferase reporter cassette was inserted between *E1A* and *E1B* open reading frames. To assess the anti-tumor efficacy, we i.v. injected UD-SCC-2 tumor-bearing NSG mice with either PBS or 2 × 10^10^ vector particles of a hexon-unmodified control vector HCRAd-5_ΔCAR or HCRAd-5-HexPos3_ΔCAR after 21 days of tumor growth. Tumor sizes were measured daily and vector-derived luciferase expression was analyzed by bioluminescence-measurement for up to 56 days after vector injection.

**Figure 5:**
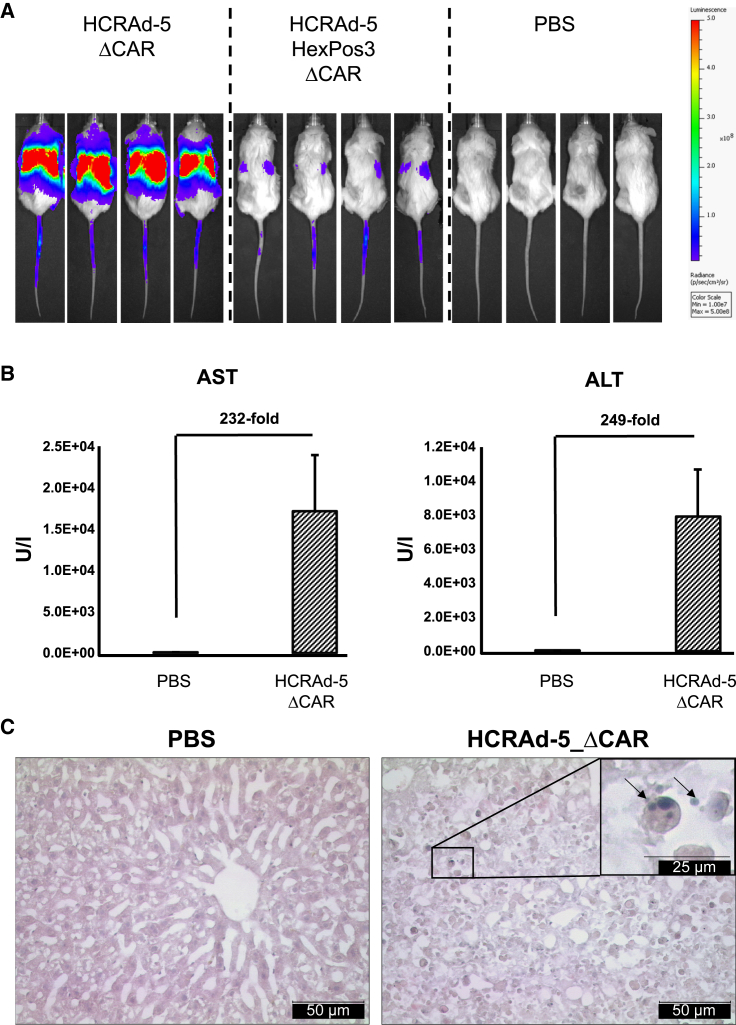
HCRAd-5_ΔCAR exhibits pronounced liver tropism and induces severe liver damage after single intravenous injection UD-SCC-2 tumor-bearing NSG mice were intravenously injected with either PBS (*n* = 12) or 2 × 10^10^ vector particles of HCRAd-5_ΔCAR (*n* = 12) or HCRAd-HexPos3_ΔCAR (*n* = 12) after 21 days of tumor growth. Two days after vector injection, (A) mice were intraperitoneally injected with 200 μL of firefly luciferase substrate under mild isoflurane narcotization and vector-derived luciferase expression was analyzed using the IVIS 200 *in vivo* imaging system. Shown are four exemplary animals per group. (B) Analysis of aspartate aminotransferase (AST) and alanine aminotransferase (ALT) serum concentrations of PBS- (*n* = 10) and HCRAd-5_ΔCAR-injected (*n* = 12) mice. (C) hematoxylin and eosin staining of liver sections of PBS- and HCRAd-5_ΔCAR-injected mice. Shown are representative pictures. PBS: well-organized liver parenchyma with a central vein and isomorphic hepatocytes. HCRAd-5_ΔCAR: swollen hepatocytes with intermingle apoptotic figures. Scale bars, 50 μm. Inset: magnified apoptotic hepatocyte with apoptotic body (arrows). Scale bar, 25 μm (inset).

Already 48 h after vector injection, we observed distinct signs of vector-induced toxicity in all HCRAd-5_ΔCAR-injected mice, as shown by an impaired activity and agility, reduced tactile stimulation, diminished skin turgor, and a rapid drop in body weight. Fitting to the biodistribution analysis (Figures 2B and 2C), we further detected strong luciferase expression at sites of the liver in these mice (Figure 5A). HCRAd-5_ΔCAR-induced toxicities necessitated the sacrifice of all respective mice already 48 h after vector injection. During final organ harvest and blood sampling, we observed internal bleedings in some of HCRAd-5_ΔCAR-injected mice and impaired coagulation in all blood samples collected. Interestingly, HCRAd-5-HexPos3_ΔCAR-injected mice did not show any signs of vector-induced toxicities and behaved like PBS-injected mice over the whole duration of the experiment. Fitting to the biodistribution analysis (Figures 2B and 2C), we also detected only minor luciferase activities at sites of the liver in all HCRAd-5-HexPos3_ΔCAR-injected mice (Figure 5A). This reconfirmed that the HexPos3 capsid mutation significantly reduced hepatocyte transduction, which is likely the reason for reduced vector toxicity.

### HCRAd-5_ΔCAR causes severe hepatic damage after i.v. injection

Since HCRAd-5_ΔCAR exhibited an abundant liver tropism and since all HCRAd-5_ΔCAR-injected mice displayed impaired blood coagulation, we hypothesized hepatic damage as the reason for the observed toxicities. Therefore, we analyzed the liver enzyme levels of aspartate aminotransferase (AST) and alanine aminotransferase (ALT) in serum samples of PBS- and HCRAd-5_ΔCAR-injected mice (Figure 5B).

AST and ALT serum concentrations in HCRAd-5_ΔCAR-injected mice were found to be elevated by 232- and 249-fold, respectively, compared with serum samples of PBS-injected mice. To confirm the suggested hepatic damage caused by HCRAd-5_ΔCAR, we histologically examined liver sections after hematoxylin and eosin (H&E) staining (Figure 5C). While liver sections of PBS-injected mice showed a well-organized liver parenchyma and isomorphic hepatocytes, we observed panlobular tissue damage and swollen hepatocytes with intermingled apoptotic figures in liver sections of HCRAd-5_ΔCAR-injected mice (Figure 5C). Thus, vector-induced toxicity of HCRAd-5_ΔCAR most likely was attributed to its pronounced liver tropism, resulting in extensive hepatocyte death and consecutive liver damage.

### A single i.v. injection of HCRAd-5-HexPos3_ΔCAR results in improved tumor transduction and slightly prolonged survival of mice

**Figure 6:**
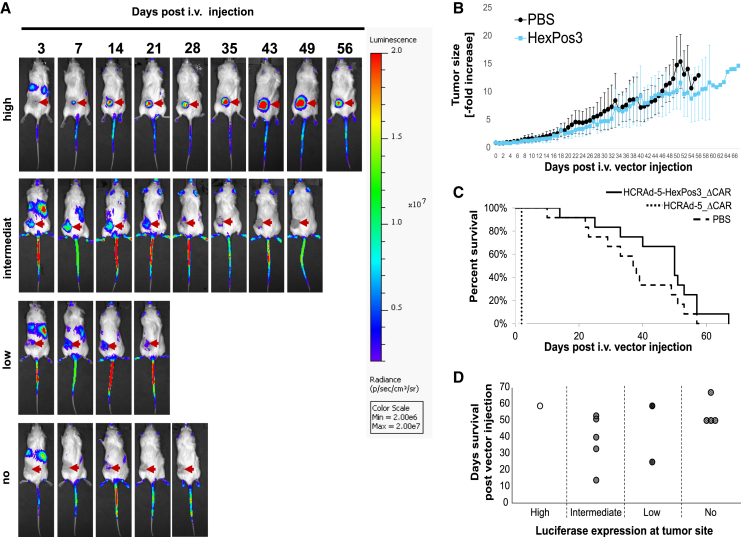
Single intravenous injection of HCRAd-5-HexPos3_ΔCAR results in successful tumor transduction and prolonged survival of UD-SCC-2 tumor-bearing NSG mice UD-SCC-2 tumor-bearing NSG mice were intravenously injected with either PBS or 2 × 10^10^ vector particles of HCRAd-5_ΔCAR or HCRAd-5-HexPos3_ΔCAR after 21 days of tumor growth. *n* = 12. (A) After 3, 7, 14, 21, 28, 35, 43, 49, and 56 days of HCRAd-5-HexPos3_ΔCAR vector injection, mice were intraperitoneally injected with 200 μL luciferase substrate under mild isoflurane narcotization and luciferase activity was analyzed using the IVIS 200 *in vivo* imaging system. According to luciferase expression levels at sites of the tumor (indicated by the red arrows), mice were grouped into “high,” “intermediate,” “low,” and “no.” Shown are representative animals of each group. (B) Tumor growth was monitored in mice injected with either PBS or HCRAd-5-HexPos3_ΔCAR. (C) Mice showing severe signs of toxicity or mice whose tumors exceeded a size of 15 mm in diameter, were sacrificed. Shown is the resulting Kaplan-Meier survival analysis of mice injected with the indicated vector. (D) Dot blot indicating time of sacrifice for each individual HCRAd-5-HexPos3_ΔCAR-injected mouse grouped into “high,” “intermediate,” “low,” or “no” luciferase expression at sites of the tumor.

HCRAd-5-HexPos3_ΔCAR was shown to exhibit significantly reduced toxicities after single i.v. injection. To further asses its biodistribution we performed bioluminescence measurements for up to 56 days after vector injection. Three days after vector administration, we detected luciferase expression at sites of the liver in all HCRAd-5-HexPos3_ΔCAR-injected mice (Figure 6A); however, this was substantially less than in HCRAd-5_ΔCAR-injected mice 2 days after vector injection (Figure 5A). Furthermore, luciferase expression at varying intensities was also detected at sites of the tumor in 8 of 12 HCRAd5-HexPos3_ΔCAR-injected mice (for representative pictures see Figure 6A), indicating successful tumor transduction. Altogether, we detected high, intermediate, low, or no luciferase expression intensities at sites of the tumor in one, five, two, and four mice, respectively. While luciferase signals at sites of the liver progressively decreased over time, i.t. luciferase expression in most of HCRAd-5-HexPos3_ΔCAR-injected mice increased up to day 7. After day 7, i.t. luciferase expression decreased for some of the animals (Figure 6A, “low” and “intermediate”). In one mouse, long-lasting and continuously increasing luciferase expression was detected for up to 56 days after vector injection (Figure 6A, “high”), suggesting i.t. vector replication.

To assess for potential anti-tumor efficacy by i.v. injected HCRAd-5-HexPos3_ΔCAR, we performed Kaplan-Meier survival analysis (Figure 6C). Accompanied with a slightly slower tumor growth (Figure 6B), HCRAd-5-HexPos3_ΔCAR-injected mice showed a slightly prolonged survival compared to PBS-injected mice (Figure 6C), although it was not statistically significant. However, a direct correlation between i.t. luciferase expression levels and prolonged survival of respective mice was not confirmed in this experiment (Figure 6D).

## Discussion

Especially after i.v. vector injection, insufficient tumor targeting and vector-induced toxicity due to off-target organ tropism limit the clinical efficacy of HAdV-5-based oncolytic vectors. Hexon, as the most abundant capsid protein, particularly the hypervariable region 1 (HVR1) within hexon, significantly contributes to the net negative surface charge of HAdV-5 particles, which in turn significantly influences its *in vivo* biodistribution profile.

We previously reported a surface charge-modified adenoviral vector that was generated by the deletion of 13 predominantly negatively charged amino acids within HVR1, which were substituted by 4 consecutive lysine residues, HAdV-5-HexPos3.^28^ We showed that the mutation significantly reduced the overall net negative surface charge of the vector particle and strongly enhanced transduction efficiencies in various cancer cell lines *in vitro*.^28^ Hypothesizing that HAdV-5-HexPos3 is a promising candidate for oncolytic virotherapy, we thoroughly characterized HAdV-5-HexPos3 regarding its oncolytic potential. Here, we show that the HexPos3 mutation results in a vector with significantly reduced off-target organ tropism, substantially reduced toxicity, and improved tumor targeting, resulting in an oncolytic vector with potentially improved overall therapeutic efficacy.

Using ΔCAR and replication-deficient vector particles, we first confirmed HAdV-5-HexPos3_ΔCAR to exhibit significantly improved transduction efficiency *in vitro* in different cancer cells lines and in a CAR-independent manner. Moreover, pre-incubation of vector particles with HAdV-5 naive NSG plasma improved transduction of different cell lines with HAdV-5-HexPos3_ΔCAR substantially. We previously reported that HAdV-5-HexPos3_ΔCAR exhibits augmented binding of blood coagulation FX compared with hexon-unmodified control vectors.^28^ Since FX is known to enhance cell transduction, this interaction might explain the here observed effect of murine plasma.^7^ We previously established a subcutaneous xenograft tumor model in immune-deficient NSG mice based on the human head and neck squamous cell carcinoma (HNSCC) cell line UD-SCC-2.^29^^,^^30^ *In vivo* i.t. injection of HAdV-5-HexPos3_ΔCAR did not result in improved tumor transduction when compared with the vector control. Since UD-SCC-2 cells were efficiently transduced *in vitro*, a possible explanation for the hindered tumor cell transduction might be a dense tumor stroma composed of cellular and extracellular matrix (ECM) components.^41^ Especially hyaluronic acid, being a dominant ECM component and a highly anionic polymer,^42^ may hinder cell infection by electrostatic interaction with the positively charged lysine residues within HVR1 of HAdV-5-HexPos3_ΔCAR. Charge-mediated vector adsorption to ECM components thus may have trapped HAdV-5-HexPos3_ΔCAR particles within the tumor stroma.

Even though HAdV-5-HexPos3_ΔCAR did not exhibit improved tumor transduction rates after i.t. injection, we performed i.v. HAdV-5-HexPos3_ΔCAR vector injection in UD-SCC-2 tumor-bearing NSG mice and analyzed its biodistribution and tumor targeting. Compared to the control vector, HAdV-5-HexPos3_ΔCAR particles were taken up significantly less into the lung, spleen, and kidney. Most importantly, HAdV-5-HexPos3_ΔCAR exhibited an almost completely ablated liver tropism. Furthermore, and in contrast to i.t. administration, HAdV-5-HexPos3_ΔCAR showed improved uptake into the tumor tissue compared with HAdV-5_ΔCAR control vectors after i.v. injection. Since we observed that NSG mouse plasma significantly enhanced cancer cell transduction by HAdV-5-HexPos3_ΔCAR *in vitro*, improved tumor cell targeting after systemic administration might be caused by interaction of particles with murine blood components. Moreover, systemically administered vector particles might infect tumor cells directly adjacent to blood vessels, thereby circumventing the ECM-derived barriers. Together with its almost completely ablated liver tropism, HAdV-5-HexPos3_ΔCAR moreover exhibited a remarkable 29-fold elevated tumor-to-liver ratio compared with HAdV-5_ΔCAR.

To ensure the safety of HAdV-5-based oAVs, another prerequisite of promising vectors is the prevention of vector replication in healthy cells. Therefore, we generated three CRAds by the introduction of defined mutations within viral regulatory genes. We consecutively introduced the previously described E1AΔ24bp,^31^ ΔE1B19k^33^ as well as the ΔE3gp19k mutation, resulting in the respective CRAds HAdV-5_E1AΔ24bp, HAdV-5_E1AΔ24bp_ΔE1B19k, and HAdV-5_E1AΔ24bp_ΔE1B19k_ΔE3gp19k and evaluated their oncolytic potential *in vitro*. Contrary to previous reports by Fueyo *et al.*,^31^ we observed the E1AΔ24bp mutation to not restrict virus replication to cancer cells, as respective HAdV-5_E1AΔ24bp lysed and spread thorough primary HSApCs with high efficiency. In contrast, and as already reported by others,^43^^,^^44^ the ΔE1B19k mutation effectively prevented replication in primary HSApCs, while it significantly accelerated replication of respective HAdV-5_E1AΔ24bp_ΔE1B19k and HAdV-5_E1AΔ24bp_ΔE1B19k_ΔE3gp19k vectors in all cancer cell lines tested. Interestingly, the deletion of 712 bp in the *ΔE3gp19k* gene deletion further accelerated the replication. Since the E3gp19k protein has no known cell-cycle modulatory functions but is involved within viral immune evasion,^38^^,^^39^^,^^40^ we speculate that the reduction in genome size might explain this observation. Thus, due to its most rapid spread in cancer cells and absent replication in primary cells, we finally considered HAdV-5_E1AΔ24bp_ΔE1B19k_ΔE3gp19k the most promising candidate as an oncolytic vector, as it potentially combined superior tumor-lytic activity and improved vector safety.

Based on the superior tumor-lytic activity and improved vector safety, HAdV-5_E1AΔ24bp_ΔE1B19k_ΔE3gp19k was chosen to be further equipped with the capsid mutations ΔCAR and HexPos3 and a luciferase expression cassette, resulting in the oncolytic vector HCRAd-5-HexPos3_ΔCAR. *In vivo* analysis in UD-SCC-2 tumor-bearing mice revealed that i.v. administration of HCRAd-5-HexPos3_ΔCAR was well tolerated, and only minor luciferase expression was detected in the liver. In contrast, mice i.v. injected with a HCRAd-5_ΔCAR control vector showed distinct signs of vector-induced toxicities already 48 h after vector injection and strong luciferase expression in the liver. Due to vector-induced toxicity of HCRAd-5_ΔCAR, all mice had to be sacrificed. Postmortem analysis indicated severe hepatotoxicity with disrupted tissue architecture, swollen and apoptotic hepatocytes, and strongly elevated AST and ALT levels in serum.^45^ Furthermore, we observed internal bleeding in some of the animals and impaired *ex vivo* coagulation of collected blood samples. Since hepatocytes reflect the main source for *de novo* synthesis of coagulation factors,^46^ most of which have a blood half-life of only a few days,^47^ the cumulative data suggest massive vector-induced liver damage in HCRAd-5_ΔCAR-injected mice. Interestingly, i.v. injection of replication-deficient HAdV-5_ΔCAR control particles at the same dose did not result in any toxicities in previous biodistribution studies, suggesting the conditionally replicating background of HCRAd-5_ΔCAR as the decisive factor for the observed hepatotoxicity. This hypothesis is supported by the finding of Engler *et al*., who reported hepatotoxicity in immune-deficient beige/SCID mice upon i.v. injection of conditionally replicating vectors, which was not observed when replication-deficient HAdV-5 vectors were injected. Reported toxicity directly correlated with viral E1A expression, genome replication, and the release of tumor necrosis factor alpha in the liver.^48^ Even though murine cells are generally non-permissive for productive human adenovirus replication,^49^^,^^50^ latent viral gene expression and genome replication may trigger cellular apoptotic pathways due to the accumulation of viral genomes,^51^ which induces cellular apoptosis.^52^^,^^53^ Excessive hepatocyte transduction thus might result in hepatocyte apoptosis, unable to be counteracted by HCRAd-5_ΔCAR due to the lack of E1B19k, which inhibits pro-apoptotic proteins.^36^ In turn, reduced hepatocyte transduction by HCRAd-5-HexPos3_ΔCAR most likely prevented such vector-induced hepatic damage and explained the absence of vector-induced toxicity.

Moreover, a single i.v. injection of HCRAd-5-HexPos3_ΔCAR resulted in successful tumor transduction; however, this did not translate into a significantly prolonged survival of respective animals compared with PBS-treated mice. However, i.t. replication and spread of HCRAd-5-HexPos3_ΔCAR was limited, as indicated by continuously decreasing i.t. luciferase expression in most of HCRAd-5-HexPos3_ΔCAR-injected mice. Fast tumor growth resulting in virus-hostile necrotic tumor cores, ECM components, and a dense tumor stroma might be causative.^54^ The vast majority of these stroma cells was most likely of murine origin. As mentioned, murine cells are generally non-permissive for human adenoviruses, therefore the murine stromal cells likely represent a virus sink and contributed to the inefficient spread of HCRAd-5-HexPos3_ΔCAR. The tumor stroma consists of various cells, with fibroblasts being the major constituent.^55^ Nilson *et al.* previously generated a conditionally replicating HAdV-5-HexPos3 vector that efficiently infected and lysed human MSCs.^30^ As MSCs share various characteristics with fibroblasts,^56^ HCRAd-5-HexPos3_ΔCAR might exhibit enhanced i.t. spread in an entirely human context as it potentially infects and lyses also stroma-associated fibroblasts more efficiently. Furthermore, although not persistent, we proved i.t. virus replication, which must result in tumor cell lysis and accompanied release of tumor-associated antigens (TAAs). In an immunocompetent setting, the virus itself and the released TAAs might induce a tumor-directed activation of the immune system, which supports the destruction of the tumor tissue.^57^ Hence, it might be that limited i.t. virus replication is sufficient to induce a strong therapeutic effect, which could not be observed in the here analyzed immunodeficient UD-SCC-2 tumor xenograft mouse model.

Taken together, HAdV-5-HexPos3 represents a promising vector platform for the generation of HAdV-5-based oncolytic viruses with reduced systemic toxicity and improved therapeutic efficacy.

## Materials and methods

### Cell lines and cell culture

A549 cells (human non-small lung carcinoma cells, ATCC, no. CCL-243) were cultivated in MEM (Gibco, no. 31095-029). UD-SCC-2 (human head and neck hypopharynx carcinoma, kindly provided by Prof. C. Brunner, Clinic for Oto-Rhino-Laryngology, University Medical Center, Ulm), UM-SCC-11B (human head and neck larynx carcinoma, kindly provided by Prof. C. Brunner, Clinic for Oto-Rhino-Laryngology, University Medical Center, Ulm), and SK-Mel-28 cells (human melanoma, ATCC no. HTB-72) were cultivated in DMEM (Gibco, no. 10938-025). N52.E6 cells^58^ (human amniocytes) were cultivated in α-MEM (Gibco, no. 22561-021). Primary HSAEpCs (PromoCell, C-12642) were cultivated in Small Airway Epithelial Cell Growth Medium (PromoCell, C-21070). DMEM, MEM, and α-MEM were supplemented with 10% fetal calf serum (FCS) (Gibco, no. 10270-106) and 1% penicillin-streptomycin-glutamine (Gibco, no. 10378-016). Small Airway Epithelial Cell Growth Medium was supplemented with Growth Medium Supplement Mix (PromoCell, C39175). If not stated otherwise, cells were routinely split twice a week, cultivated at 37°C, 5% CO_2_ and 90% relative humidity, further referred to as standard conditions.

### Adenoviral vectors

All vectors used in this study were based on HAdV-5 of species C (GenBank: AY339865.1). Genetic modifications were introduced using homologous recombination (Red/ET Recombination; Gene Bridges, no. K002) according to the manufacture’s protocol.

All replication-deficient adenoviral vectors were *E1*-deleted first-generation vectors (Δnt 441–3,522). All *E1*-deleted vectors contained, in the E1 region, an eGFP reporter cassette under hCMV promoter control (subcloned from pEGFP-N1; Clontech).

Since interaction of the adenoviral fiber with CAR does not influence biodistribution in mice,^13^ but binds erythrocytes in a human setting,^59^ all vectors used for *in vivo* assays were additionally ablated for CAR binding (ΔCAR). These vectors harbored a single amino acid substitution within the DE loop of the fiber knob domain (GenBank: AAQ19310.1, Y477A).

Vectors harboring the HexPos3 capsid mutation have been published before^28^ and harbored a defined 13 amino acid deletion within the HVR1 of hexon, which was replaced by 4 consecutive lysine residues (GenBank: AY339865.1; nt 19,280–19,318; EEEDDDNEDEVDE → KKKK).

HAdV-5wt and all CRAds were based on the wild-type adenovirus genome except for indicated modifications of early gene regions. CRAds with the E1AΔ24bp mutation harbored a 24 bp deletion within the CR1 of *E1A* (Δ nt 919–943). CRAds with the ΔE1B19k mutation harbored a 147 bp deletion within the *E1B* gene region (Δ nt 1,770–1,916). CRAds with the ΔE3gp19k mutation harbored a 712 bp deletion within the *E3* gene region (Δ nt 28,738–29,450). In addition, the start codon of the E3gp19k protein at position nt 28,729 was mutated (nt 28,729–28,732 ATGA → ATAA) to maintain the stop codon of the upstream-located E3-7.1K protein.

HAdV-5wt and CRAds additionally contained a hCMV promoter-driven eGFP (subcloned from pEGFP-N1; Clontech) and NanoLuciferase (subcloned from pNL1.1. plasmid, N109A, Promega) fusion reporter gene or a hCMV promoter-driven firefly luciferase (GenBank: MK484107.1) reporter gene inserted forward between the *E1A* and *E1B* open reading frames at position nt 1,648.

### Adenoviral vector purification

All replication-deficient, *E1*-deleted adenoviral vectors were propagated in *E1*-transcomplementing N52.E6 cells.^58^ Replication competent HAdV-5wt and CRAds were propagated in A549 cells following transfection with respective purified and linearized viral DNA. All vectors and viruses were purified as described previously.^28^ In brief, 4 × 10^8^ cells were infected with respective virus or vectors, harvested 48 h post transduction and resuspended in 3 mL resuspension buffer (50 mM 4-[2-hydroxyethyl]-1-piperazineethanesulfonic acid [HEPES], 150 mM NaCl [pH 7.4]). Cells, infected with HexPos3 capsid-mutated vectors, were resuspended in HEPES buffer containing 250 mM NaCl to avoid charge-mediated binding of vector particles to cell debris. Resuspended cells were lysed by three consecutive freeze-thaw cycles and cell debris was separated by centrifugation at 2,000 × *g* for 15 min. In the case of HexPos3 capsid-mutated vectors, cell lysates were first incubated with 5 units of benzonase at 37°C for 30 min to digest free nucleic acids prior to centrifugation. As a next step, vector particles were purified by two-step CsCl gradient ultracentrifugation. First, vector particle-containing cell lysate supernatant was added on top of a CsCl step-gradient (bottom: 1.41 g/mL; top: 1.27 g/mL dissolved in 50 mM HEPES, 150 mM NaCl [pH 7.4]) and centrifuged at 176,000 × *g* for 2 h at 4°C. Vector particles were aspirated from the gradient and loaded onto a second continuous CsCl gradient (1.34 g/mL dissolved in 50 mM HEPES, 150 mM NaCl [pH 7.4]), followed by centrifugation at 176,000 × *g* for 20 h at 4°C. Vector particles were aspirated from the gradient and desalted by size-exclusion chromatography using PD10 columns (GE Healthcare, 17-0851-01). Physical vector titers were determined using optical density measurement at 260 nm.^60^

For all produced vectors, genome integrity was verified by sequencing and restriction enzyme analyses and the purity of vector samples was confirmed by SDS-PAGE with subsequent silver staining of separated proteins (data not shown).

### *In vitro* cell transduction and infection

A total of 2 × 10^4^ cells/well were seeded in 96-well flat-bottom plates (Thermo Fisher Scientific, no. 167008) and cultivated under standard conditions overnight. After 24 h, supernatants were aspirated, cells were washed once with PBS and 100 μL of respective serum-free cell culture medium was added per well. Subsequently, cells were transduced/infected in triplicates with the indicated pMOI of respective vectors or virus diluted in 100 μL serum-free cell culture medium. For transduction assays using murine plasma, vectors were diluted in 2 μL PBS, mixed with 10 μL NSG plasma and incubated at 37°C for 10 min. Pre-incubated vector particles were subsequently diluted to a final volume of 100 μL in serum-free cell culture medium and added to the cells. After 3–4 h incubation under standard conditions, virus/vector-containing supernatants were aspirated, cells were washed once with PBS and 200 μL of respective serum-containing cell culture medium was added per well. For flow cytometric analyses, wells were detached 24 h post transduction/infection and analyzed for eGFP-positive cells and mean fluorescence intensity. For microscopic evaluation of virus replication and spread through the cell layers, eGFP-positive cells were visualized 10 days post infection using the Leica DM IL fluorescence microscope.

### Flow cytometric analysis

Cells, transduced with eGFP-encoding HAdV-5 vectors were washed once with PBS and detached with TrypLE select. Subsequently, cells were thoroughly mixed with an equal volume of PBS + 0.5% FCS and analyzed by flow cytometry using a CytoFlex Cytometer (Beckmann Coulter).

### Assessment of cytotoxicity by crystal violet staining

A549, UD-SCC-2, SK-Mel-28, and HSAEpC cells were seeded onto Nunclon Delta-coated, flat-bottom 96-well plates at a density of 2 ×1 0^4^ cells/well in 200 μL serous medium. The next day, 100 μL cell culture medium was removed from each well and substituted by 100 μL fresh serum-containing cell culture medium containing vector or virus particles at the indicated pMOI. Cells were cultivated under standard conditions for 7–14 days, depending on the respective cell line used (see Figure 4). After the indicated time, supernatants were removed, and cells were washed twice with 200 μL PBS. Next, cells were fixed with 100 μL/well of 4% PFA (dissolved in PBS) at room temperature for 15 min and subsequently washed with 200 μL PBS. Remaining cells were stained with 50 μL/well of 0.1% crystal violet solution (dissolved in ddH_2_O) for ∼2 min. Subsequently, cells were washed three times with 200 μL PBS and air-dried before pictures were taken.

### Animals

NSG mice were purchased from the laboratory animal facility at Ulm University. Mice were kept in pathogen-free, individually ventilated cages and fed with sterilized diet for laboratory rodents. All animal experiments were performed according to the policies and procedures of the institutional guidelines and were approved by the Animal Care Commission of the Government Baden-Württemberg (TVA, no. 1433).

### Plasma and serum preparation

For plasma samples, whole-blood samples were anticoagulated with 1 μg/μL Argatroban (Sigma-Aldrich, A0487-5MG) dissolved in PBS and centrifuged at 800 × *g* for 10 min to remove blood cells including platelets, and cleared plasma fractions were transferred to new tubes. For serum samples, whole blood was coagulated for 30 min at room temperature before serum supernatants were transferred to new tubes. Plasma and serum samples were stored at −20°C.

### Establishment of human tumor xenografts

UD-SCC-2 cells (2 × 10^6^) were diluted in 50 μL PBS and mixed with 50 μL ice-cold growth factor-reduced Matrigel (Merck, no. CLS356252) prior to subcutaneous injection into the left flank of NSG mice. Xenografts were grown for 14, 21, or 28 days or until tumors reached a critical size of 15 mm in diameter.

### *In vivo* biodistribution and tumor transduction experiments

For biodistribution and tumor-targeting studies, UD-SCC-2 tumor-bearing female NSG mice between age 8 and 12 weeks were used. For i.v. injection, 2 × 10^10^ replication-deficient vector particles were dissolved in 100 μL PBS and injected into the tail vein after 14 days of tumor growth. For i.t. injection, 1 × 10^10^ vector particles, dissolved in 50 μL PBS, were injected after 28 days of tumor growth via three radial dispersed injection routes into the tumor. Three days after vector injection, mice were euthanized by overdosed Sevoflurane narcotization with analgesia using 5 mg/kg carprofen subcutaneously. Animals were perfused using PBS, and subsequently organs and tumors were harvested and snap-frozen in liquid nitrogen. In addition, parts of liver tissues were fixed in 2% PFA (dissolved in PBS) at 4°C for 24 h, followed by tissue dehydration in 20% sucrose (dissolved in ddH_2_O) at 4°C for an additional 24 h. Fixed and dehydrated liver samples were finally embedded into TissueTek and frozen at −80°C for subsequent preparation of cryo-sections.

### Determination of liver enzymes

Determination of ALT and AST in murine serum samples was performed using assays from Roche Diagnostics. These *in vitro* tests are used for the quantitative determination of ALT or AST with pyridoxal phosphate activation on the cobas c system 503 and follow the recommendations of the International Federation of Clinical Chemistry and Laboratory Medicine. For highly elevated liver values, murine serum samples were diluted 1:10 in sodium chloride.

### Survival studies

For survival studies, UD-SCC-2 tumor-bearing male and female NSG mice between age 6 and 8 months were used. After 21 days of tumor growth, mice were injected into the tail vein with either PBS or 2 × 10^10^ vector particles of either HCRAd-5_ΔCAR or HCRAd-5-HexPos3_ΔCAR dissolved in 100 μL PBS. Tumor growth was monitored by daily measurements of tumor volumes using the formula V_(tumor)_ = 0.5 × tumor length × (tumor width)^2^. Mice were sacrificed when tumors reached a critical size of 15 mm in diameter or when animals showed signs of toxicity defined by a score sheet incorporating, e.g., reduction of body weight, apathy, or stop grooming.

### *In vivo* bioluminescence imaging

Mice, i.v. injected with either PBS or firefly luciferase encoding HCRAd-5_ΔCAR and HCRAd-5-hexPos3_ΔCAR, were intraperitoneally injected with 300 μL VivoGlo luciferin (10 mg/mL dissolved in PBS) and narcotized by mild isoflurane anesthesia, while eyes were protected with eye ointment. Narcotized mice were subsequently transferred into the IVIS 200 imaging system chamber and pictures were taken at different exposure times (30 s to 5 min) using the following settings: binning 4; F/stop 4; field of view 4; subject height 1.5 cm. Bioluminescence measurement of PBS- and HCRAd-5_ΔCAR-injected mice was performed once 48 h post vector injection. Mice, injected with HCRAd-5-HexPos3_ΔCAR were additionally analyzed 3, 7, 14, 21, 28, 35, 43, 49, and 56 days post vector injection.

### Fluorometric analysis of tissue homogenates

Thawed tissue was added to 200 μL suspension buffer (50 mM Tris, 150 mM NaCl [pH 7.4]), supplemented with 1× protease inhibitor (Roche, no. 118735800001), and homogenized using a bullet blender. The homogenate was centrifuged twice at 20,817 × *g* for 5 min, while after each centrifugation step the obtained cleared middle phase was transferred into a new 1.5 mL reaction tube. The cleared sample was diluted 1:10 in suspension buffer and total protein concentration was determined using optical density measurement at a wavelength of 280 nm. Fluorescence intensities of 2 μL samples were determined using the NanoDrop 3300 device at an excitation and emission wavelength of 488 and 512 nm, respectively. Results were calculated as relative fluorescence units per μg protein.

### Genomic DNA extraction from tissue samples

Genomic DNA extraction from snap-frozen tissue samples was performed using the GenElute Mammalian Genomic DNA Miniprep Kit (Sigma-Aldrich, no. G1N350-1KT), according to the manufacturer. Purified DNA was eluted in 10 mM Tris (pH 8.0) and DNA concentrations were determined by optical density measurement at a wavelength of 260 nm.^60^

### Quantitative real-time PCR

Twenty nanograms of purified genomic DNA in 2 μL was mixed with 10 μL SYBR Green (Kapa Biosystems, no. KK4502) and 0.4 μL of forward and reverse primer each (10 pmol/μL) in a final volume of 20 μL. Thermo protocol: initial heat at 95°C for 10 min (1 cycle), denaturation at 90°C for 30 s, annealing at 60°C for 30 s, elongation at 72°C for 8 s (40 cycles), final elongation at 72°C for 2 min (1 cycle), storage at 4°C. For standardization, detected adenoviral genome copy numbers were normalized to either human or murine β-actin genome copy numbers in 2 μL total DNA of the same sample. Primer used for detection of the adenoviral *E4* region: forward: 5′-TAGACGATCCCTACTGTACG-3′; reverse: 5′-GGAAATATGACTACGTCCGG-3′. Primers used for detection of human β-actin: forward: 5′-GCTCCTCCTGAGCGCAAG-3′; reverse: 5′-CATCTGCTGGAAGGTGGACA-3′. Primers used for detection of murine β-actin: forward: 5′-CAAGGAGTGCAAGAACACAG-3′; reverse: 5′-GCCTTGGAGTGTGTATTGAG-3′.

### Fluorescence microscopy of liver sections

PFA-fixed and TissueTek-embedded frozen liver samples were cut to a thickness of 6 μm using a Leica cryostat microtome. Sections were covered with fluorescence mounting medium (Dako, no. S3023) and analyzed for eGFP-positive cells by fluorescence microscopy.

### H&E staining of tissue sections

Six-micrometer cryo-sections of PFA-fixed and TissueTek-embedded frozen liver samples were fixed in ice-cold acetone and air dried. Sections were incubated in Mayer’s hematoxylin solution for 3 min and washed with tap water for 10 min. Next, sections were stained with eosin solution (0.5% in ddH_2_O) for 1 min and incubated in ddH_2_O for 10 s. Subsequently, sections were dehydrated by incubation in 70%, 96%, and 100% EtOH for 30 s, 1 min, and 4 min, respectively. Lastly, sections were incubated in Xylol for 4 min and covered with Eukitt for microscopic evaluation.

### Statistical analysis

If not stated otherwise, all experiments were repeated at least three times. Statistical analyses were conducted using RStudio Software (version 4.1.2) using either unpaired two-sample (Welch’s) Student’s t test or Wilcoxon test. Statistical significance was considered for *p* values ≤0.05 and results are given as mean ± standard deviation.

## Data and code availability

Data are contained and available within this manuscript.

## Acknowledgments

We thank Prof. Cornelia Brunner from the University Medical Center Ulm for providing the HNSCC cell lines. Furthermore, the authors acknowledge Bernd Baumann from the Institute of Physiological Chemistry at Ulm University for the support regarding allocation and use of the bioluminescence measurement. The work was supported by the 10.13039/501100002347German Federal Ministry of Education and Research (BMBF) and the Federal States of Germany Grant “Innovative Hochschule” (FKZ 3IHS024D).

## Author contributions

Conceptualization and methodology, F.W., S.K., and L.K.; investigation, F.W., R.N., E.A., S.P., T.F.E.B., and L.K.; project administration, F.W., S.K., and L.K.; writing – original draft, F.W. and L.K.; writing – review & editing, S.P., S.K., R.N., T.F.E.B., and L.K.; data discussion and evaluation, F.W., R.N., T.F.E.B., S.K., and L.K.; funding acquisition, S.K.

## Declaration of interests

Ulm University has filed a patent application on the subject of this manuscript, with authors R.N., S.K., and L.K. named as inventors.
